# Comparison of the oxidative reactivity of recombinant fetal and adult human hemoglobin: implications for the design of hemoglobin-based oxygen carriers

**DOI:** 10.1042/BSR20180370

**Published:** 2018-07-03

**Authors:** Michelle Simons, Svetlana Gretton, Gary G.A. Silkstone, Badri S. Rajagopal, Victoria Allen-Baume, Natalie Syrett, Thoufieq Shaik, Nelida Leiva-Eriksson, Luca Ronda, Andrea Mozzarelli, Michael B. Strader, Abdu I. Alayash, Brandon J. Reeder, Chris E. Cooper

**Affiliations:** 1 School of Biological Sciences, University of Essex, Wivenhoe Park, Colchester, Essex CO4 3SQ, United Kingdom; 2 Department of Pure and Applied Biochemistry Lund University, Box 124, Lund 221 00, Sweden; 3 Department of Medicine and Surgery, University of Parma, Parma, Italy; 4 Department of Food and Drug, University of Parma, Parma, Italy and Institute of Biophysics, National Research Council (CNR), Pisa, Italy; 5 Laboratory of Biochemistry and Vascular Biology, Center for Biologics Evaluation and Research, Food and Drug Administration, Silver Spring, MD, U.S.A.

**Keywords:** adult, blood substitute, fetal, hemoglobin, oxidative stress, oxygen carrier

## Abstract

Hemoglobin (Hb)-based oxygen carriers (HBOCs) have been engineered to replace or augment the oxygen carrying capacity of erythrocytes. However, clinical results have generally been disappointing, in part due to the intrinsic oxidative toxicity of Hb. The most common HBOC starting material is adult human or bovine Hb. However, it has been suggested that fetal Hb may offer advantages due to decreased oxidative reactivity. Large-scale manufacturing of HBOC will likely and ultimately require recombinant sources of human proteins. We, therefore, directly compared the functional properties and oxidative reactivity of recombinant fetal (rHbF) and recombinant adult (rHbA) Hb. rHbA and rHbF produced similar yields of purified functional protein. No differences were seen in the two proteins in: autoxidation rate; the rate of hydrogen peroxide reaction; NO scavenging dioxygenase activity; and the NO producing nitrite reductase activity. The rHbF protein was: less damaged by low levels of hydrogen peroxide; less damaging when added to human umbilical vein endothelial cells (HUVEC) in the ferric form; and had a slower rate of intrinsic heme loss. The rHbA protein was: more readily reducible by plasma antioxidants such as ascorbate in both the reactive ferryl and ferric states; less readily damaged by lipid peroxides; and less damaging to phosphatidylcholine liposomes. In conclusion in terms of oxidative reactivity, there are advantages and disadvantages to the use of rHbA or rHbF as the basis for an effective HBOC.

## Introduction

Globally, there is a need for a sustainable, long-lasting, artificial blood substitute due to increased demand, the risk from new blood-borne diseases, difficulty in matching rare blood types, and religious aversion to blood products [1]. There is also a growing realization that at a lower dose these products can facilitate oxygen delivery to compromised tissue even in the presence of adequate numbers of red blood cells. In these cases, the products are acting not as providers of oxygen, but as facilitators of oxygen transferred from the red cells to the hypoxic tissue. These so-called ‘oxygen therapeutics’ have been suggested to be of benefit in a variety of pathologies such as trauma, sickle cell disease, and subarachnoid hemorrhage [2].

The use of the oxygen carrying hemoglobin (Hb) is favored as a starting material for such an extracellular oxygen delivery product; consequently the field of Hb-based oxygen carriers (HBOCs) has resulted in a wide variety of preclinical and clinical trials in this area, although with rather limited current success [3]. Traditionally, Hb is purified from animal erythrocytes (including human), removing or reducing the stroma content; this Hb is then treated to increase its size and vascular retention rate by cross-linking, polymerization, conjugation, or encapsulation [4]. Although these approaches may increase protein stability, they also have the potential to increase product heterogeneity and to alter reactivity [5].

The starting material for Hb production has varied. Bovine Hb has been used due to ease of production [6], similar to human Hb and potentially favorable antioxidative properties [7]. Whereas marine worms produce large Hb polymers (erythrocruorins) that have naturally evolved to function outside the cellular environment and hence can be used as an HBOC without any post-purification modifications [8]. However, the most common animal source for HBOC is human. Although historically outdated banked blood has been favored, recombinant techniques have also been used [9]. Indeed it is hard to see how a bulk product such as HBOC could ultimately be produced worldwide from discarded material from human blood banks, especially given the drive to decrease waste and hence the volume of outdated blood available as a source of Hb [10]. Recombinant human Hb has been produced in many species including pigs [11], yeast [12], mice, and *Escherichia coli* [13–15]. Somatogen took HBOC product (rHb 1.0) made from *E. coli* and produced human Hb in clinical trials [16].

Baxter Healthcare subsequently bought Somatogen ensuring a long-term production platform for its HBOC products combined with the additional flexibility of engineering favorable properties into the product [9]. The resultant Baxter HBOC product (rHb2.0) entered clinical trials, but was discontinued due to adverse side reactions triggered by extracellular Hb [9]. Extracellular Hb toxicity derives from a combination of nitric oxide scavenging reactions and an intrinsic oxidative toxicity, which can lead to damage of cells, proteins, lipids, and DNA in the surrounding areas [17]. Free heme released from unstable Hb molecules also has the potential to activate the immune system [18,19] by acting as a damage-associated molecule pattern molecule (DAMP). Using recombinant tools, it is possible to design a protein to be less oxidatively reactive [20]. It is also possible to engineer decreased nitric oxide scavenging properties, decreasing the vasoconstriction produced by extracellular Hb [21]. However, these mutations can sometimes come at the cost of a reduced Hb stability and an increased heme loss with associated toxic side effects [9,20], issues that likely led to the discontinuation of rHb2.0.

HBOC products have largely focussed on using the adult HbA-based molecule. However, HbA is not the only form of human Hb. Human Hb is a tetramer of homologous protein dimers of α- and β-type subunits. α-like genes are encoded on chromosome 16 (α, ζ) and β-like genes on chromosome 11 (β, γ, δ, ε). During development, the Hb tetramer changes from embryonic form of Hb to the fetal and then adult forms. The development lifetimes overlap, but is broadly in the order: HbE Gower-1 (ζ_2_ε_2_); HbE Portland-1 (ζ_2_γ_2_); HbE Gower-2 (α_2_ε_2_); HbF (α_2_γ_2_); and HbA (α_2_β_2_). These proteins have different functional properties suitable for their developmental roles.

The HbF molecule has recently proved to be of interest in HBOC design. Differences in the N-terminus between the β and γ subunits affect tetramer’s stability and gives HbF tetramers a 70-fold higher affinity compared with HbA, increasing the tetramer/dimer ratio [22,23]. It has therefore been suggested that introducing mutations to make the β subunit more like the γ subunit might be beneficial in the production of a recombinant HBOC; some success has been achieved using this approach [14]. However, there is nothing more γ-like than the γ subunit itself. Therefore, HbF makes a better starting material for HBOC production than HbA [24]. HbF might also benefit from a decrease in Hb oxidative properties seen in recent studies [25,26]. However, matched against this is historical data suggesting that HbF could be more pro-oxidative than HbA [27] and the emerging view that the oxidative properties of HbF are implicated in damage in the maternal circulation in pre-eclampsia [28].

We therefore decided to do a detailed side-by-side comparison of the properties of HbF and HbA produced by identical recombinant methods, focussing in particular on those reactions likely to be implicated in adverse side reactions when these products are incorporated into an HBOC [3].

## Methods

### Protein expression and purification

*E. coli* BL21 (DE3) cells transformed with pETDuet plasmid containing genes for the adult or fetal wild-type Hb were grown in 2 l Erlenmeyer flasks containing 1.4 l growth medium at 37°C, and agitated at 180 rpm until an OD_600_ ~2 was achieved. Hb expression was then induced by the addition of 0.5 mM IPTG, 0.25 mM ALA (aminolevulinic acid), and 0.1 mM ferric citrate. Cultures were then bubbled with pure CO gas, flasks sealed thoroughly with rubber bungs, and grown for a further 18 h at 30°C at 90 rpm. Cells were then harvested by centrifugation at 3500 ***g*** for 20 min at 4°C.

Our previous rHb purification method used cation exchange and size exclusion methodology [29]. A small, variable quantity contaminating protein ~25 kDa was always found using this method. This has previously been attributed to covalently linked dimers of β-Hb subunits [30]. The presence of only β subunits in our ~25 kDa contaminant was confirmed by MS, suggesting a similar assignment (data not shown). The addition of anion exchange and metal affinity chromatography (details below) to the previous cation exchange resulted in almost complete removal of the contaminant, resulting in >95% purity.

Cell pellets were resuspended in 10 mM sodium phosphate buffer, pH 6.0, before being lysed using an Avestin C3 Emulsiflex homogenizer. The cell lysate was cleared by centrifugation at 38000 ***g*** for 30 min at 4°C and the pH was adjusted to 6.0 by the addition of small quantities of orthophosphoric acid. The centrifugation step was then repeated and the lysate filtered through a 0.45-µm syringe filter before being loaded on to a CM Sepharose column (GE Healthcare) pre-equilibrated with 10 mM sodium phosphate buffer, pH 6.0. Hb was then eluted with a gradient of 70 mM sodium phosphate, pH 7.2. Selected fractions were then pooled and the volume decreased in a spin concentrator and loaded on to a 5 ml Q-HP column (GE Healthcare) pre-equilibrated with 70 mM sodium phosphate buffer, pH 7.2. Protein was eluted with a 100 ml gradient of 50 mM sodium phosphate, pH 7.2, and 100 mM sodium chloride. Fractions containing Hb were pooled and applied to a 5 ml HisTrap HP column (GE Healthcare) and eluted with a 100-ml gradient of 50 mM sodium phosphate, pH 7.2, 100 mM sodium chloride, and 100 mM imidazole. Finally, Hb was concentrated using an Amicon Ultra 30000 MWCO (Millipore) and then buffer exchanged into 70 mM sodium phosphate, pH 7.2. All buffers used during the purification procedure were bubbled with CO and the lysate and proteins were bubbled with CO at every stage. Four to fifteen percent of gradient TGX stain free precast gels (Bio-Rad) were used to assay purity following each column stage. The concentration of the ferrous CO (carbonmonoxyHb) bound form of Hb was calculated using an extinction coefficient at 419 nm of 191000 M^−1^.cm^−1^ [31] and stored in liquid nitrogen.

### Optical spectroscopy

Unless stated otherwise, all optical spectra were taken using a Cary 5000 spectrophotometer (Agilent). To produce the various different forms of Hb, the carbonmonoxyHb was first oxidized to the metHb (ferric Fe^3+^) form by the addition of excess potassium ferricyanide, while under constant illumination under a strong but low temperature light source that acts to photolyze the CO from the ferrous iron. The ferricyanide was then removed by gel filtration down a PD-10 column (GE Healthcare). The deoxyHb form was made by addition of a slight excess of sodium dithionite to the metHb form. This was then applied to a PD-10 column to generate oxyHb.

### p50 measurements

The p50 measurements were carried out diluting concentrated stock Hb solutions in 100 mM HEPES buffer, pH 7.0, 100 mM sodium chloride, 1.2 mM sodium phosphate, and 1 mM EDTA. The final protein concentration was 100 µM. Before dilution, the samples were centrifuged to remove precipitates.

Oxygen equilibrium curves were measured at 25°C, as previously reported [32]. For each sample, the absorption spectrum (equilibrated in air) was collected immediately after thawing. The Hayashi enzymatic reducing system was added to the solution before titrations to reduce metHb and to limit its formation during titration. Following, the samples were deoxygenated using a helium flow and then equilibrated with different oxygen partial pressures.

### Autoxidation

Autoxidation of 10 µM oxyHb in 70 mM sodium phosphate buffer, pH 7.2, with 12.5 mU superoxide dismutase (SOD) and 10 nM catalase, at 37°C, was monitored optically. The oxyHb concentrations were calculated using the extinction coefficients of 125000 (415 nm) and 14600 (577 nm) using the units M^−1^.cm^−1^ [31]. Time courses (406–500 nm) were fitted to single exponential function.

### Heme release

The metHb (4 µM) was incubated with hemopexin (4.5 µM) obtained from human plasma (Sigma) in 70 mM sodium phosphate buffer, pH 7.2, at 37°C. The concentration of the metHb proteins were calculated optically using the extinction coefficient of 184000 M^−1^.cm^−1^ at 405 nm for the H_2_O bound high-spin form at pH ~7. The time courses (401–418 nm) were fitted to double exponential functions.

### Comparative analysis of oxidative hotspots in HbA and HbF using quantitative proteomics

HbA and HbF proteins were treated with incremental doses (0, 5×, 10×) of H_2_O_2_ and incubated overnight in 20 mM sodium phosphate buffer, pH 7.4 at 25°C. All samples were processed and trypsinized using standard procedures. LC-MS/MS analysis and targetted quantitative proteomics were utilized to identify and quantitate oxidative modifications in both Hb samples. All data were performed in triplicate on a Q-Exactive mass spectrometer (Thermo Scientific) coupled to a Proxeon HPLC system. MS/MS spectra were initially searched against the human database supplemented with the above recombinant sequences using both Protalizer software (Vulcan Analytical) and the Mascot database search algorithm (Matrix Science) to confirm sequence identity and identify unmodified and oxidized version of ‘hot spot’ peptides; all oxidized peptide reproducibly identified from database searches were then quantitated. Extracted ion chromatograms (XICs) of modified (and unmodified) tryptic peptides were used to quantitate oxidative differences. For relative quantitation, the ratio of each oxidized ‘hot spot’ peptide was calculated based on the sum of the XIC peak area of all forms (oxidized and unmodified) to be 100%. Hotspot residues used for oxidative comparisons included only conserved residues found on β and γ. All other ‘hotspot’ containing peptides identified in the present study were consistent with previously published data.

### Ferryl formation by reaction with peroxide

Varying concentrations of H_2_O_2_ were reacted with 10 µM metHb in a rapid mixing stopped-flow diode array spectrophotometer (Applied Photophyiscs) in 10 mM sodium phosphate buffer, pH 7.2 at 37°C. For each concentration of H_2_O_2_, three reactions were carried out, and the time courses generated averaged to a single rate trace and then analyzed. The time courses (552–630 nm) were fitted to double exponential functions using the fitting program incorporated within the instrument. The contribution of the absorbance for both phases and for all the kinetic traces generated was approximately the same. The two rate constants were then plotted against H_2_O_2_ concentration and straight lines of best fit applied to the data points.

### Ferryl reduction by ascorbate

MetHb (10 µM) in 70 mM sodium phosphate buffer, pH 7.2, was reacted with 30 µM H_2_O_2_ at 25°C for ~10 min to produce ferrylHb (Fe^4+^). The reaction of metHb proteins with H_2_O_2_ was monitored optically to ensure 100% ferryl formation. Trace catalase was added to remove excess H_2_O_2_ and this was incubated for a few seconds to fully react. Sodium ascorbate was then added in a 1:1 volume ratio so that final concentration of Hb was 5 µM. The reactions were then followed to completion, optically, using an Agilent 8453 diode array spectrophotometer. The time courses (425–406 nm) were fitted to double exponential functions minimizing the least squares using Microsoft Excel Solver. For each time course, the two calculated rate constants were assigned to the reactions of the α and β/γ subunits, and the data plotted as a function of reductant concentration for each protein. This ascorbate concentration dependent kinetic profile was fitted to a rectangular hyperbola plus a straight line (α subunits) or a single rectangular hyperbola (β/γ subunits).

### Ferric reduction by ascorbate

MetHb (20 µM) in 70 mM sodium phosphate buffer, pH 7.2, was reacted with a 1:1 volume ratio with sodium ascorbate at 25°C so that the final concentration of Hb was 10 µM and ascorbate was 0.1 or 10 mM. The time courses (577–630 nm) were fitted to a single exponential function.

### Nitrite reductase

The proteins were made and maintained in their deoxy states by addition of an excess of the reductant sodium dithionite to metHb. DeoxyHb (10 µM) was reacted with sodium nitrite of varying concentrations in 70 mM sodium phosphate buffer, pH 7.2 at 24°C. The reactions were monitored optically using an Agilent 8453 diode array spectrophotometer. The time courses (432–413 nm) were fitted to a single exponential function minimizing the least squares using Microsoft Excel Solver. The data were plotted as a function of nitrite concentration for each protein and the second order rate constants were determined by fitting to a straight line.

### NO scavenging

An NO solution was prepared by dissolving the NO donor ProliNONOate (Cayman Chemical Company) in 25 mM NaOH. The concentration of ProliNONOate was determined using an extinction coefficient of 8500 M^−1^.cm^−1^ at 250 nm (when added to the buffer at neutral pH, 1.8 molecules of NO are released per ProliNONOate molecule). The ProliNONOate was then diluted into thoroughly degassed 70 mM sodium phosphate buffer, pH 7.2. 10 μM oxyHb was reacted 1:1 with varying concentrations of ProliNONOate in a rapid mixing stopped-flow spectrophotometer (Applied Photophyiscs). Due to the speed of the reaction, the temperature was set to 15°C. Reactions were monitored at 422 nm and fitted to a single exponential. The data were plotted as a function of NO concentration for each protein and the second order rate constants were determined by fitting to a straight line.

### Lipid oxidation

α-phosphatidylcholine (5 mg/ml) derived from soybean (Type II-S, Sigma) in 10 mM sodium phosphate buffer, pH 7.2, was sonicated in a water bath for ~5 min until no particulates could be seen. To produce unilamellar liposomes of uniform diameter, this suspension was then passed through a Northern Lipids liposome extruder containing a membrane with size cutoff 100 nm (Whatman) for a minimum of ten times. Liposomes were stored at 4°C and used within 4 h.

MetHb or oxyHb (2 µM) was incubated with 200 µg/ml liposomes, for 10 h at 25°C. Sodium ascorbate was added at a final concentration of 50 µM as indicated. Lipid oxidation was monitored by following the production of conjugated dienes using a Tecan Infinite M200Pro plate reader. The time courses (234–650 nm) were plotted and used to determine the lag time before the onset of lipid oxidation.

### Reactions with 13S-hydroperoxy-9Z,11E-octadecadienoic acid

The lipid hydroperoxide 13S-hydroperoxy-9Z,11E-octadecadienoic acid (HPODE) was produced as previously described [33]. HPODE (50 μM) was reacted with 3.7 μM metHb in a rapid mixing stopped-flow spectrophotometer (Applied Photophyiscs) in 50 mM sodium phosphate buffer, pH 7.2 at 37°C in the presence or absence of 50 μM ascorbate. With the instrument in the photomultiplier/monochromator set up, heme bleaching was monitored using a single wavelength at 405 nm. The rate traces were fitted to a double exponential function (the amplitudes of the two phases were constrained to be equal) using the fitting program incorporated within the instrument.

### Primary cell culture, treatment, and assays

Human umbilical vein endothelial cells (HUVECs), a primary cell line, was obtained from Lonza. Cells were maintained in complete primary cell EGM medium (Lonza) in a humidified atmosphere with 5% CO_2_ at 37°C until seven passages were reached. For experiments to test the effect of rHb on cells, cells were trypsinized, counted, plated into 24-well plates and maintained until 70% confluent.

Prior to all treatments, cells were gently washed with warm PBS and the medium was replaced by serum and phenol-free EGM medium for HUVECs. Cells were treated by adding either 50 µM Hb (recombinant adult Hb (rHbA) and recombinant fetal Hb (rHbF) in oxy or met forms), 50 µM ascorbate, or 10 mU/ml glucose/glucose oxidase system (GOX) to the medium in the total volume of 250 µl. Cells were placed back into the incubator and harvested after either 4 or 24 h as indicated. All experimental conditions were performed in four replicates.

MetHb proteins were prepared as previously stated. The highly concentrated oxyHb proteins required for addition to the cells were prepared by incubating carbonmonoxyHb under bright constant illumination while gently blowing a stream of pure oxygen over the surface of the liquid, on ice. Conversion into oxyHb form was monitored spectrally and was completed by ~30–45 min.

Cellular damage was assessed by measuring levels of lactate dehydrogenase (LDH) released into cellular medium. The LDH assay (Sigma–Aldrich) was performed after 24 h of exposure to the experimental treatment according to manufacturer’s instructions. The absorbance at two wavelengths (450 and 650 nm) was obtained using a Tecan Infinite M200Pro plate reader and both values were used to calculate levels of LDH.

The cellular response to oxidative stress in treated cells was evaluated by activity of SOD. The SOD assay (Sod OxySelect superoxide dismutase activity assay kit (Cell Biolabs Inc.)) was performed according to manufacturer’s instructions 4 h post treatment on lysed cells. The absorbance values at 490 and 650 nm were taken and the level of SOD activity calculated.

### Statistics

HbA was compared with HbF under the same experimental conditions. Data are presented as either mean and SD of *n* replicate experiments (statistical significance determined via the Student’s *t* test) or mean and S.E.M. derived from non-linear curve fitting of *n* points in Kaleidagraph version 4.5.2 (statistical significance determined via the lack of overlap of 95% confidence intervals of means). The specific test used for each measurement is indicated in Table 1 and/or in the relevant figure legend.

**Table 1:** Differences between rHbA and rHbF

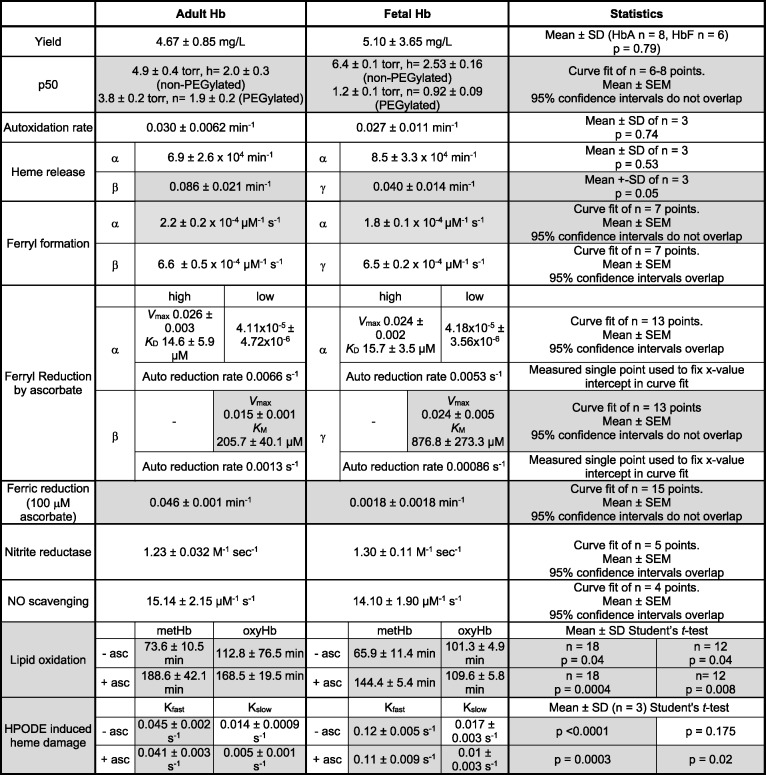

Statistically significant results comparing HbA with HbF are highlighted in gray. Data are presented as either mean ± S.D. of *n* replicate experiments (statistical significance determined via the Student’s *t-*test) or the mean ± S.E.M. derived from curve fitting of *n* points (statistical significance determined via the overlap or not of 95% confidence intervals of the means). Yield is calculated as milligrams of purified protein per liter of bacterial culture; for p50 results, h indicates the Hill coefficient; for lipid oxidation and HPODE damage results −/+ asc indicates −/+ 50 μM ascorbate.

## Results

The results of all experiments performed to compare rHbA and rHbF are summarized in Table 1. All protein concentrations are given in µM of the total heme present.

### Yield

rHbA and rHbF were expressed and purified as described in ‘Methods’ section. Gel electrophoresis was used to confirm purity of Hb (Supplementary Figure S1). The HPLC of the two recombinant proteins (Supplementary Figure S2) shows that no significant amount of protoporphyrin-IX was present as can occur with lack of iron. We did not observe any statistical differences in the yields between rHbA and rHbF (Table 1). However, there was considerable variability in batch to batch yield, particularly for rHbF.

### Oxygen binding

Oxygen binding curves were measured for our preparations of rHbA and rHbF. Analysis of these curves allowed calculation of the p50 and Hill coefficient values [32]. Our results showed that both rHbA and rHbF can bind oxygen in a co-operative manner but that rHbF has a slightly lower affinity and slightly higher co-operativity (Hill coefficient) compared with rHbA (Table 1 and Supplementary Figure S3). Upon PEGylation the p50 and Hill coefficients of both proteins decreased, but more so for rHbF resulting in a relatively high affinity but low co-operativity for the PEGylated product.

### Autoxidation and heme loss

The autoxidation of HbA and HbF was measured at 37°C for a 3-h period in the presence of SOD and catalase. The time course (406–500 nm) was fitted to a single exponential; the rates of autoxidation of rHbA and rHbF were not significantly different (Table 1). Heme loss from the met form of rHbA and rHbF was measured at 37°C for a 10-h period. The resulting kinetic traces (401–418 nm) could be fitted to a double exponential function with the two rates attributed to either the α/β or α/γ subunits for rHbA and rHbF, respectively. The slower rate of heme loss in rHbA was assigned to the α subunit and the faster rate to the β subunit as determined from previous studies of heme dissociation kinetics [34–36]. The slower of the two rates was not statistically different between rHbA and rHbF and therefore the slower rate of heme loss in rHbF was also assigned to the α subunit. In contrast, the faster rates for rHbA and rHbF, assigned to the β and γ subunits respectively, were significantly different; slower for rHbF compared with rHbA (Table 1).

### Ferryl formation and reduction

**Figure 1:**
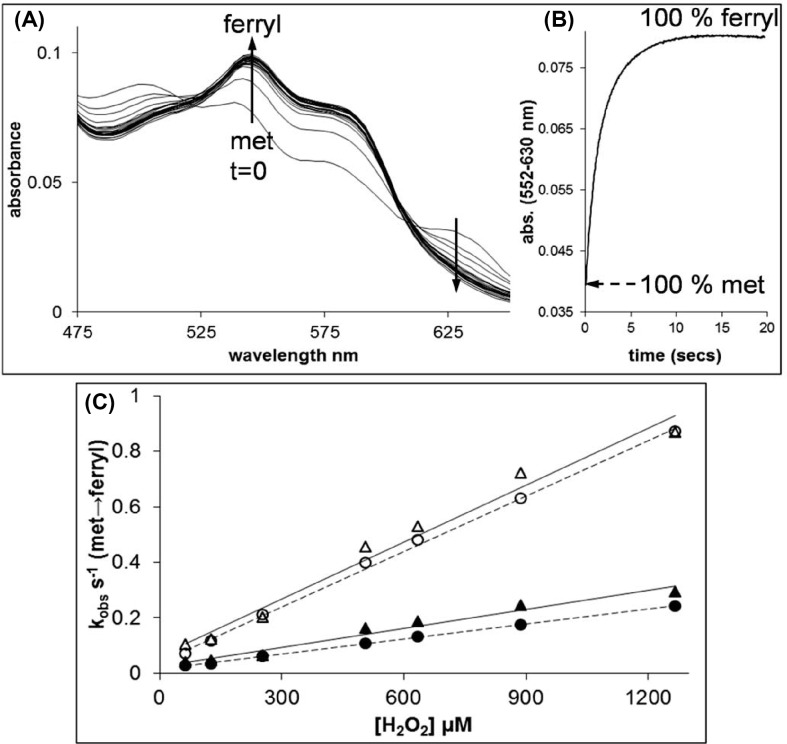
Rates of ferryl formation measured by the reaction of H_2_O_2_ with the met forms of rHbA and rHbF The met forms of rHbA and rHbF (10 µM) were reacted with varying amounts of H_2_O_2_ in 20 mM sodium phosphate buffer, pH 7.2 at 37°C. (**A**) The spectral changes observed in the visible region for the conversion of met rHbF into the ferryl form on reaction with 1 mM H_2_O_2_. Spectra are shown every ~1 s of the reaction from t = 0 to 20 s. (**B**) A typical kinetic trace for the conversion of met rHbA into the ferryl form on reaction with 1 mM H_2_O_2_. (**C**). The observed rate constants for the reactions of rHbA and rHbF with varying concentrations of H_2_O_2_. For rHbA and rHbF, the *k*_obs_.s^−1^ value was calculated by fitting the kinetic traces to double exponential functions. Triangles = rHbA (open = fast, closed = slow), circles = rHbF (open = fast, closed = slow). Straight lines of best fit have been applied to the datasets.

The rates of ferryl formation of rHbA and rHbF were measured via the reactions of the ferric Hb with hydrogen peroxide. On rapid mixing of peroxide with metHb forms of rHbA and rHbF spectral changes were observed consistent with the conversion of the metHb into the ferrylHb forms (Figure 1A). The kinetic traces for the reaction (552–630 nm) confirmed that the ferryl species was fully formed at all the peroxide concentrations used (Figure 1B). The time courses were fitted to a double exponential function and the rates attributed to the α subunits (slower rate) or β/γ subunits (faster rate) and plotted against peroxide concentration (Figure 1C). These data showed a linear dependence on the concentration of H_2_O_2_ and the second-order rate constants for the conversion of metHb into ferrylHb were calculated (Table 1). There was no difference observed between adult and fetal Hb fast rates (β/γ subunits), and while there is a small statistical difference in the slow rates (α subunits) it is not likely to be meaningful.

**Figure 2:**
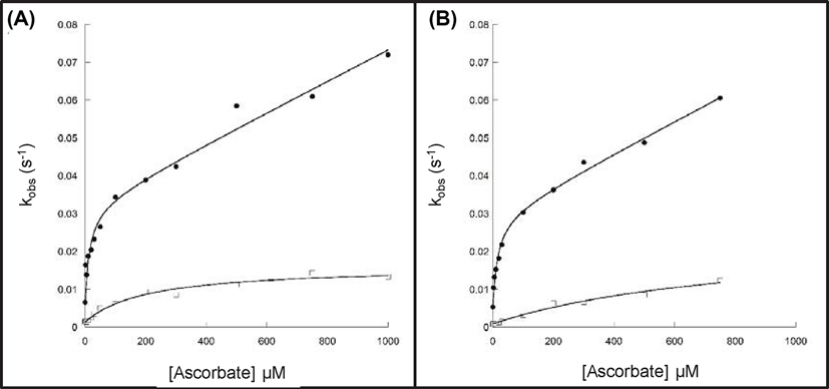
Dependence of the rate constants for ferryl Hb reduction on ascorbate concentration for adult and fetal Hb Ferryl Hb (10 µM) was reacted with ascorbate in 70 mM sodium phosphate buffer, pH 7.2 and rate constants for the two phases of ferryl reduction were determined by fitting to a double exponential function. (**A**) rHbA and (**B**) rHbF. Closed symbols represent α subunit (the rate constants were fitted to a rectangular hyperbola plus a straight line function (solid lines)), and open symbols represent β/γ subunit (fitted to a rectangular hyperbola (solid lines)).

The ferryl reduction kinetics of rHbA and rHbF were compared in the presence of differing concentrations of ascorbate. The time courses for the reduction were fitted to double exponential functions and the two rates assigned to the α or β/γ subunits of rHb. There was a slight decrease in the autoreduction rate for rHbF compared with rHbA (Table 1). The kinetics of ferrylHb reduction as a function of ascorbate concentration are presented in Figure 2. The faster rate constant (α subunit) could be best fitted to a rectangular hyperbola plus a straight line, as the second phase of the curve did not conform to a hyperbolic function. These two phases correspond to direct reduction of the heme (low affinity) and electron transfer via surface exposed tyrosine residues (high affinity) [29]. The slower rate constant (β/γ subunit) only had a single slow phase, corresponding to the low affinity pathway, and was fit to a single rectangular hyperbola. The values for both the high and low affinity pathways for the α subunits of rHbA and rHbF are comparable (Table 1). The values for the low affinity pathway for the β/γ subunits are different. rHbF has 1.5-times higher *V*_max_ than rHbA. However, this is offset by a four times higher *K*_M_, meaning that at low (physiological) levels of ascorbate the ferryl iron in the β subunit is more readily reduced by ascorbate than ferryl iron in the γ subunit.

### Ferric reduction

**Figure 3:**
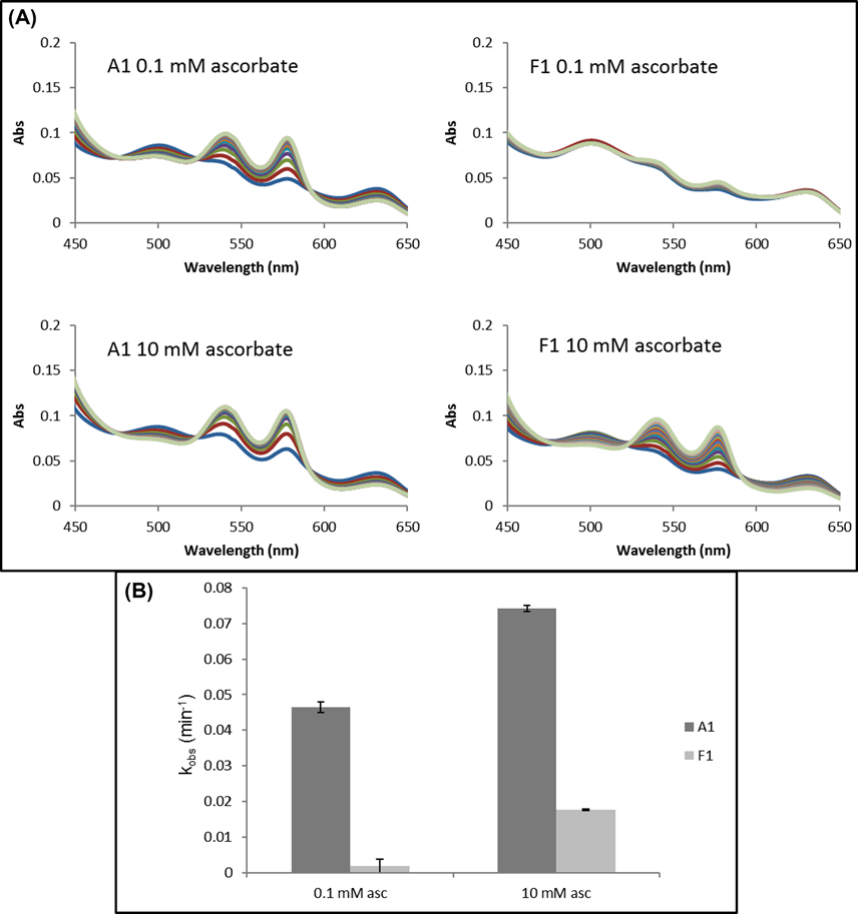
Ferric reduction by ascorbate for adult and fetal Hb Ferric (met) Hb (10 µM) was reacted with ascorbate in 70 mM sodium phosphate buffer, pH 7.2 and reduction of metHb to oxyHb monitored optically and rate constants were determined by fitting to a single exponential function. (**A**) Example of absorbance spectra under conditions indicated. (**B**) Rates of ferric reduction at high and low concentrations of ascorbate for A1 (dark gray) and F1 (light gray).

The ferric reduction kinetics of rHbA and rHbF were measured by reaction of metHb to oxyHb with high and low ascorbate concentrations. Spectral changes were observed consistent with the conversion of the metHb into the oxyHb forms (Figure 3A). The time courses (577–630 nm) were fitted to a single exponential function and at both ascorbate concentrations used rHbA had a greatly increased rate of ferric reduction compared with rHbF (Figure 3B and Table 1).

### Nitrite reductase

**Figure 4:**
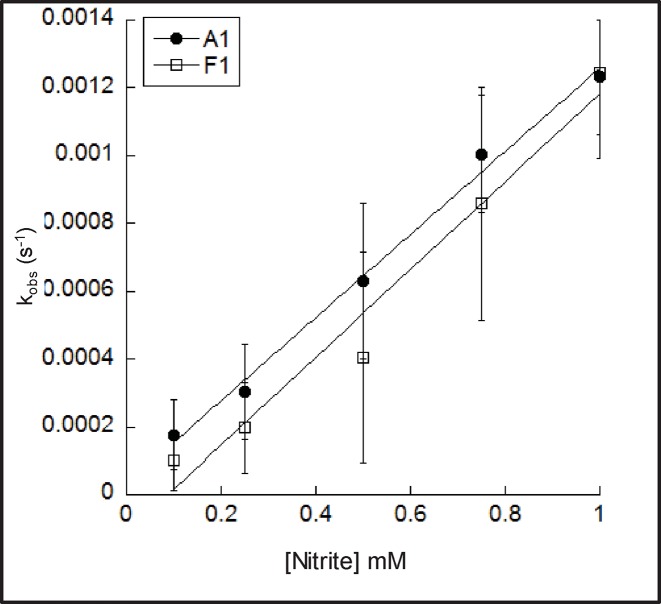
Nitrite reductase activity of adult and fetal Hb DeoxyHb (10 µM) was reacted with sodium nitrite in 70 mM sodium phosphate buffer, pH 7.2. Rate constants were determined by fitting to a single exponential function and were plotted against nitrite concentration. The second order rate constants were then determined by fitting to a straight line (filled circles represent rHbA and open squares rHbF).

The nitrite reductase activities of rHbA and rHbF were compared by measuring the reaction of deoxyHb with sodium nitrite. The resulting kinetic traces (432–413 nm) were fitted to a single exponential and plotted against nitrite concentration (Figure 4). These data were fit to a straight line; the second order rate constants for rHbA and rHbF were not statistically different (Table 1).

### Nitric oxide dioxygenase (NO scavenging)

**Figure 5:**
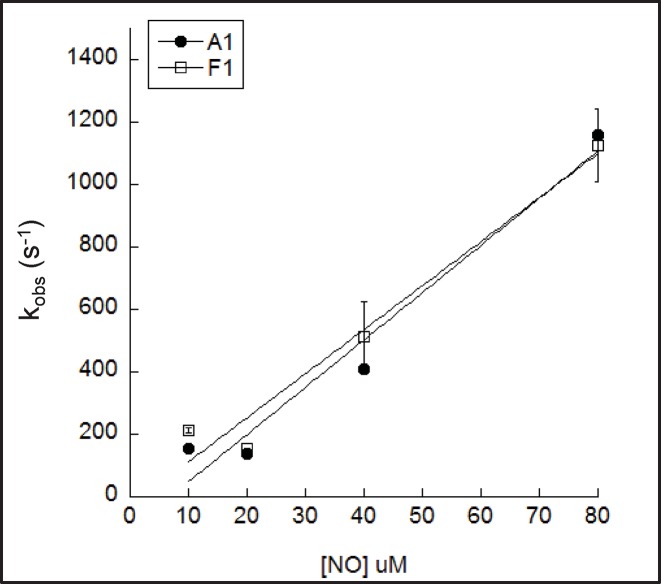
NO scavenging activity of adult and fetal Hb OxyHb (5 µM) was reacted with NO in 70 mM sodium phosphate buffer, pH 7.2 at 15°C. Rate constants were determined by fitting to a single exponential function and were plotted against NO concentration. The second order rate constants were then determined by fitting to a straight line (filled circles represent rHbA and open squares rHbF).

The NO dioxygenase activity of rHbA and rHbF was compared by measuring the conversion of oxyHb into metHb at varying concentrations of NO. The resulting kinetic traces (422 nm) were fitted to a single exponential function and plotted against NO concentration (Figure 5). These data were fitted to a straight line; the second order rate constants for rHbA and rHbF were not statistically different (Table 1).

### Comparative analysis of oxidative hotspots in HbA and HbF using quantitative proteomics

Experimental oxidative conditions were performed to test the role H_2_O_2_ has on the observed post-translational oxidation of oxidative hotspots in recombinant HbA and HbF. Toward this goal, we utilized quantitative MS to target all peptide charge states. XICs were generated from the most abundant monoisotopic peak of each peptide isotopic profile and the resulting ratio differences were compared for oxidized and unoxidized hotspot peptides.

As purified (Table 2) β/γ C93 tri-oxidation of HbF was slightly higher than HbA (6.3% compared with 3.17%). In both cases, the extent of oxidation correlated with incremental H_2_O_2_ increases. For example, 10× H_2_O_2_ resulted in nearly a ten-fold higher ratio for HbA C93 oxidation and five-fold higher ratio for HbF compared with the control where no peroxide was added. The data in Table 2 also show tri-oxidation of αC104. For both proteins, α104 cysteic acid oxidation levels were at consistently lower levels than β/γ C93 tri-oxidation; rHbA showing higher oxidation than rHbF at baseline, but appearing more resistant to added peroxide.

**Table 2:** H_2_O_2_ induced tri-oxidation of C93 and C104 of rHbA and rHbF

H_2_O_2_:Hb	rHbA βC93	rHbF γC93	rHbA αC104	rHbF αC104
0	3.17 ± 0.22%	6.3 ± 0.81%	1.9 ± 0.79%	0.8 ± 0.21%
5:1	21.5 ± 1.48%	20.9 ± 0.38%	1.1 ± 0.17%	1.28 ± 0.16%
10:1	29.7 ± 0.25%	28.9 ± 0.79%	1.1 ± 0.07%	2.3 ± 0.15%

Data are presented as mean ± S.D. of *n*=3 replicate experiments.

### Oxidative damage to lipids and primary cells

#### Liposome reactions

**Figure 6:**
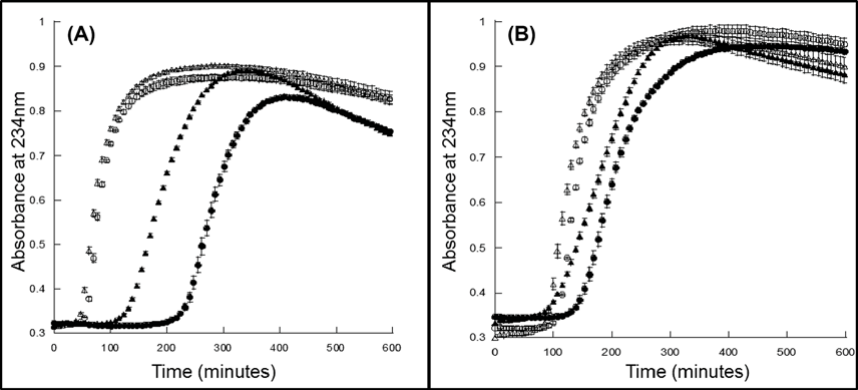
Lipid oxidation lag times for rHbA and rHbF rHb (2 µM) was reacted with liposomes in 70 mM sodium phosphate buffer, pH 7.2, ±50 µM ascorbate, and production of conjugated diene formation was measured via absorbance at 234 nm. The lag time before oxidation was calculated. (**A**) MetHb and (**B**) oxyHb. rHbA circles, rHbF triangles, open symbols without reductant, filled symbols with 50 µM ascorbate. Values are the mean from three measurements and error bars represent S.D. from the mean.

The lipid oxidation of the metHb and oxyHb forms of rHbA and rHbF with and without reductant was compared (Figure 6). In the absence of reductant, the lag phase of the met form of rHbF was significantly shorter than that of rHbA. When reductant was added (50 µM ascorbate) the lag times greatly increased but the lag time of rHbF was still shorter than rHbA (Table 1 and Figure 6A). Similarly for the oxyHb form rHbF had statistically significantly shorter lag times. In the presence of reductant, the lag times did not increase as dramatically for the oxyHb form as for the metHb form but rHbF still produced shorter lag times (Table 1 and Figure 6B).

#### HPODE reactions

**Figure 7:**
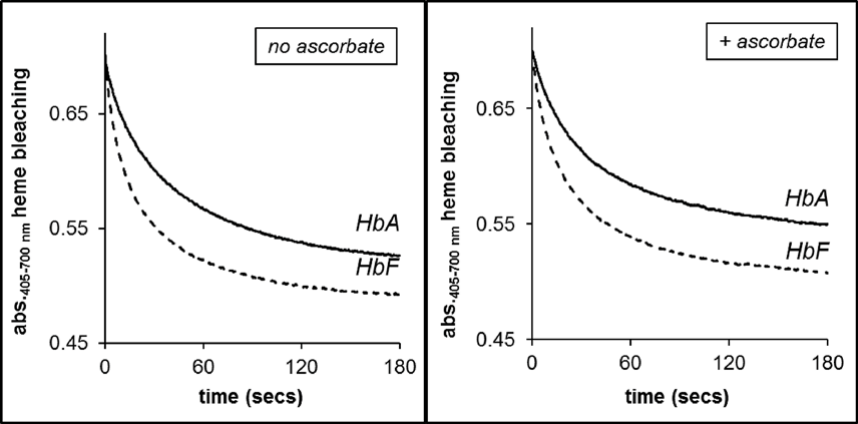
The reactions of the met forms of rHbA and rHbF with HPODE, in the absence and presence of the physiological reductant ascorbate HPODE (50 μM) was reacted with 3.7 μM metHb in a rapid mixing stopped-flow spectrophotometer in 50 mM sodium phosphate buffer, pH 7.2 at 37°C ± 50 μM ascorbate. Rate traces showing heme bleaching over time measured at 405 nm.

Figure 7 shows the reactions of the metHb forms of rHbA and rHbF at a fixed concentration of 50 µM of the lipid peroxide HPODE in the absence or presence of reductant. The time course of heme bleaching was fitted to a double exponential function. Both in the absence and presence of reductant the fast rates for rHbF were found to be significantly faster than rHbA. The difference between the slow rates however was found to be not statistically significant. Addition of the reductant (ascorbate) resulted in a minor decrease in the reactivity of both proteins with HPODE.

#### Primary cells

**Figure 8:**
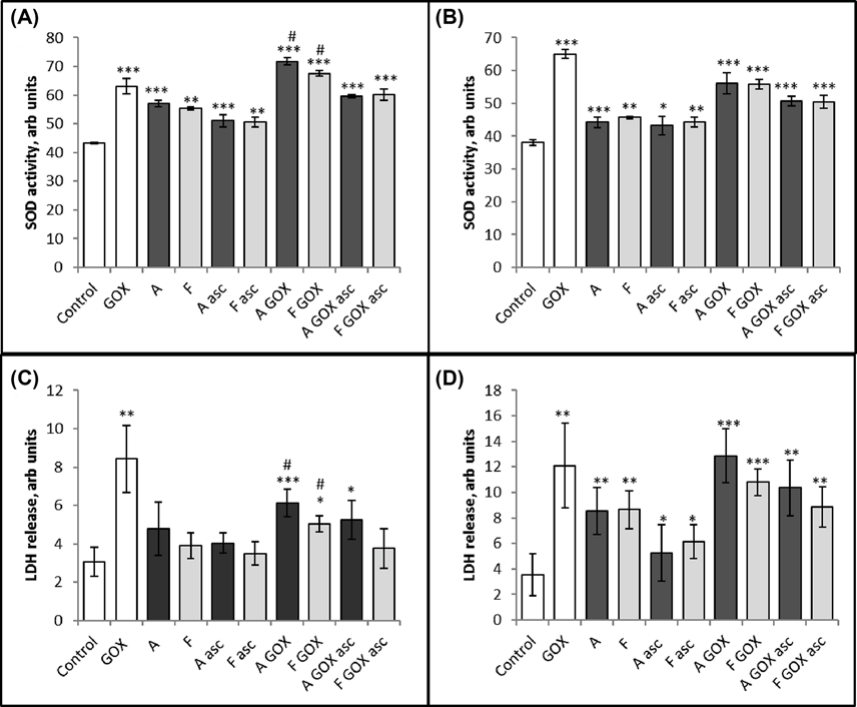
The effects of adding rHbA (**A**) and rHbF (**F**) to HUVECs HUVECs were incubated in serum and phenol-free EGM medium containing either 10 mM sodium phosphate buffer, pH 7.2 (control), 10 mU/ml GOX or 50 µM rHbA and rHbF metHb (**A**,**C**) and oxyHb (**B**,**D**) in the absence or presence of reductant (ascorbate). For the SOD assay (A,B), cells were lysed 4 h post treatment. Cellular medium was used for LDH assay (C,D) 24 h post treatment. Each bar represents mean ± S.D. of four experimental repeats. Statistical differences between control and treatment conditions were analyzed using unpaired Student’s *t*test and are presented as * where *P<0.05, **<0.01, ***<0.001. Statistical differences between rHbA and rHbF under each condition are presented in Supplementary Table S1; ^#^ represents *P*<0.05.

Cell viability due to cellular membrane damage was measured (24 h post treatment) by LDH release, while short term (4 h post treatment) oxidative stress was measured by SOD induction. Hydrogen peroxide addition (via a GOX) was used as a positive control and caused significant increases in both assays (Figure 8). Varying the concentration of rHb established that a 50 μM concentration was optimally sensitive in our assays (data not shown).

Different redox states of rHbA and rHbF were then tested. Figure 8 shows the cytotoxic effects of rHbA and rHbF in combination with 10 mU GOX and 50 µM ascorbate (as indicated) on HUVECs. The cells were lysed after 4 h for the SOD assay and the cellular medium was taken for the LDH assay 24 h post treatment. There was increased oxidative stress and membrane damage following the addition of adult and fetal rHb compared with control cells (significance indicated in Figure 8). In general, combination rHb with GOX increased the cytotoxic damage, compared with treatment with Hb alone, whereas, addition of 50 µM ascorbate to the cellular medium rescued the cytotoxicity of rHb, but not to the level of control cells. In general, there were no significant differences between rHbA and rHbF in the majority of the treatments (Supplementary Table S1). However, rHbA did show a somewhat larger increase in both SOD induction and LDH release when met and oxy forms were added to cells in the presence of GOX alone.

## Discussion

Our detailed comparison of rHbA and rHbF revealed some differences that could affect their ability to be an effective component of an HBOC.

### Preparation and oxygen-binding properties

Using our expression system we were able to produce similar yields of adult and fetal Hb in shake flasks, not inconsistent with historical studies [37]. However, in order to manufacture Hb in large enough quantities for an HBOC, production would need to shift to large-scale fermentation. This has been achieved for HbA [38,39], but not yet for HbF.

*In vivo*, HbF has a higher oxygen affinity than HbA in order to enable the fetus to capture oxygen from the maternal circulation. However, in contrast with some organisms, in humans this difference is not an intrinsic property of the functional tetramer, but instead by a well-characterized modulation of the binding of the physiological effector molecule bisphosphoglycerate (BPG) [40]. This is therefore less likely to be relevant when Hb is used outside the red blood cell as an HBOC. Consistent with this and previous studies [37], we observed only a small difference between the two proteins under ‘pseudo-physiological’ conditions intended to mimic plasma rather than red blood cells (pH 7.0, 100 mM sodium chloride, 1.2 mM sodium phosphate). Any small ‘intrinsic’ oxygen p50 differences between the use of the γ and β subunit is anyway likely to be only minor compared with subsequent treatments of the protein. To form a viable HBOC, Hb needs to be modified to remain longer in the vasculature and these treatments have significant effects on the oxygen affinity. In the present study, we used the EURO-PEG-Hb method that aims to preserve a more normal oxygen affinity by PEGylating the protein in the deoxy T state [41]. In this case, we did see more significant differences between the proteins. Following EURO-PEG PEGylation protocol, fetal Hb had a higher oxygen affinity than the adult protein and lacked co-operative oxygen binding (Supplementary Figure S3). This higher oxygen affinity for PEGylated fetal Hb is not necessarily a bad property. Sangart have previously championed MP4 (Hemospan) – a high affinity Hb with no co-operative oxygen binding – as oxygen therapeutic. However, the p50 for EURO-PEG-HbF is even lower than the 5 Torr for MP4, making it only able to deliver oxygen to the most hypoxic tissues. Therefore, the addition of mutations to raise the p50 might be necessary.

### Protein stability – autoxidation, heme loss, and heme reduction

Outside the red blood cell, oxyHb is oxidized easily to metHb which is physiologically inactive [42]. MetHb is also far more likely to lose its heme cofactor than oxyHb [36]. Given the ferric:superoxide (Fe^3+^−O_2_^•−^) nature of the iron-oxygen bond in oxyHb, all else being equal, one might expect the oxygen affinity to vary inversely with the rate of autoxidation in globins with very high oxygen binding affinity corresponding to low autoxidation. This relationship does indeed hold for a variety of mutations in the myoglobin heme pocket that stabilize or destabilize the Fe^3+^−O_2_^•−^ bond; mutations that stabilize the bond increase oxygen affinity and decrease autoxidation [43]. Intriguingly, the opposite relationship (a linear correlation between oxygen affinity and autoxidation) exists if the heme group itself is modified to shift the resonance state between Fe^2+^−O_2_ and Fe^3+^−O_2_^•−^. A heme modification that shifts the resonance toward the Fe^2+^−O_2_ form results in an increase in both the O_2_ affinity and a decrease in autoxidation [44]. Understanding (and potentially decreasing) the autoxidation rate is therefore an important factor in HBOC design as it can impact both oxygen affinity and oxidatively damaging properties. In this context, we found that rHbA and rHbF had the same intrinsic rate of autoxidation, mirroring their very similar oxygen affinities.

The rate of heme release [36] from Hb is a major consideration while making an HBOC. Heme can trigger oxidative reactivity itself, but perhaps more importantly acts as a DAMP molecule activating Toll-like receptor 4 (TLR4) of the innate immune system leading to oxidant production, inflammation, and vascular injury. High amounts of autoxidation and free heme release from the recombinant protein rHb3011 likely led to the failure of HBOC derived from it in clinical trials [9]. Heme is preferentially released from the ferric (met) form of Hb. Perhaps unsurprisingly our results indicated that the α subunits of both proteins have the same rate of heme release from the ferric state. However, the slower rate of heme release from the γ subunits compared with the β indicates that in this respect rHbF is more stable than rHbA.

Outside the cell, the dominant reaction regenerating oxyHb from metHb is the non-enzymatic reduction by plasma ascorbate [45]. Reduction of metHb by ascorbate is known to occur via preferential reaction of the β subunits [46]. Our results with rHbF show that the same is not true for the γ subunit with the rate of reduction in γ being as slow as the α subunit. Therefore, rHbF reduction from metHb to oxyHb is very slow in comparison with rHbA even at high ascorbate concentrations.

In summary, both proteins have advantages and disadvantages. The oxy forms autoxidize at a similar rate. The resultant metHb is more readily reduced back to oxyHb for the adult protein. However, as long as it remains in the ferric state, the adult protein is more likely to lose its heme cofactor.

### Formation and reduction of ferryl heme

Once autoxidized to Fe^3+^, the resultant metHb can be further oxidized by peroxide(s) to the Fe^4+^ ferryl species. Lacking protective catalase, in the plasma levels of peroxide can increase under stress conditions such as tissue injury; therefore, extracellular Hb is potentially more susceptible to damage by peroxide and ferryl formation. The ferryl Hb species is known to be damaging *in vivo* [3,47], generating lipid oxidation products such as 4-hydroxynonenal and isoprostanes, both biomarkers of oxidative stress and in the case of isoprostanes, potent vasoactive compounds [48,49]. It would be a desired property of any potential blood substitute to have either a slow rate of ferryl formation or once ferryl is formed, for it to be quickly converted into the less damaging ferric form. Our comparison of the recombinant proteins found no statistical differences in the rates of ferryl formation by reaction with peroxide. This is in contrast with that shown for native Hb where the HbF γ chain was oxidized at a slightly higher rate than HbA β chain [26].

Once ferryl Hb has been formed, the ferryl heme can be reduced to the met form by a range of compounds and reductants [29,33,50,51]. It has previously been shown that this occurs via two distinct pathways of electron transfer from the reductant to the heme iron: direct reduction of the heme (low affinity) and electron transfer via surface exposed tyrosine residues (high affinity) [29,47,52]. Our comparison of rHbA and rHbF found no difference in reduction in the α subunits via the high or low affinity pathways. However, the *K*_M_ for the low affinity pathway of the β/γ subunits was four times higher for fetal than adult meaning rHbF is less able to remove ferryl Hb via reduction by ascorbate. As it is possible to engineer increases in this rate as desired by the insertion of redox active tyrosine residues into the β subunit [29,47,50] in adult proteins, it is likely a similar effect could be engineered into the γ subunit to overcome this deficit.

### Nitric oxide reactivity

The NO dioxygenase activity of oxyHb (oxidation to met) means that cell-free Hb rapidly scavenges NO, causing vasoconstriction and an increase in blood pressure [21,53]. Our results showed no difference in adult and fetal Hb NO oxidation. Mutations to introduce bulky amino acid residues to limit ligand entry into the pocket have produced Hb molecules with lower NO scavenging and blood pressure effects [9]. There is no reason to suppose that homologous mutations in fetal Hb would not have the same effect.

Nitrite reduction by deoxyHb generates NO, initially binding to deoxyHb but eventually being released as NO gas resulting in vasodilation [54–56]. Thus the nitrite reductase activity of Hb has been suggested to be a useful property for an HBOC to possess, overcoming the decrease in NO levels caused by the oxidation reaction [57]. Indeed nitrite is sometimes added following HBOC addition, though with varying levels of success [58]. We saw no difference in the rate of nitrite reduction of deoxyHb between the recombinant adult and fetal proteins in the absence of oxygen (hence with Hb in the T state). This is perhaps somewhat surprising given the faster reactivity seen in the reaction of native T state fetal compared with adult ovine Hb [59]. However, as the fastest – and more likely physiologically relevant reactions are seen in the R state protein [60,61] – the lack of a difference here may not have relevant consequences for HBOC design.

### Ease of oxidation of amino acid subunits

Previously it has been shown that βC93 is the prominent end point for free radical induced protein oxidation; oxidation at other residues (β, γ, or α) is usually lower [62]. Our data agree that the α subunit is more resistant to oxidation and therefore more oxidatively stable in the Hb tetramer. The γ chain appeared somewhat more resistant than the β chain as in the absence of added peroxide rHbF showed less oxidation than rHbA. However, both proteins responded to incremental increases in peroxide in a similar manner and at higher peroxide this fetal/adult difference disappeared, suggesting the proteins are broadly equivalent in terms of oxidative stability. In our recombinant proteins, we did not see the dramatic preferential increase in stability for native fetal Hb compared with native adult Hb observed previously [26]. It is unclear whether this difference is a fundamental feature of native compared with recombinantly produced proteins or instead due to differences in the purification methods used. On the one hand, it is sometimes difficult to remove contaminating antioxidant proteins (catalase/SOD) from Hb purified from red blood cells and on the other hand oxidation can more readily occur in the production and purification of recombinant proteins. Either effect could cause the experimental differences observed between the present paper and previous studies [26].

### Oxidative damage to lipids

It is not clear what implication differences in amino acid oxidizability have for HBOC. The amino acids in fetal Hb seem less likely to be oxidized by peroxide; yet fetal Hb reacts as fast with peroxide and the ferryl and ferric states are less readily detoxified by reductants. Oxidants have to go somewhere; it could be argued that, in sacrificing its own amino acids and heme, the adult protein is less likely to damage external biomolecules. On the other hand, the features of fetal Hb (decreased Hb amino acid oxidation and decreased reactivity to reductants) mirror the effects of adding haptoglobin to Hb which is known to protect against Hb-induced oxidative damage such as lipid oxidation [63]. We therefore tested directly the ability of rHbA and rHbF to react with external oxidized external lipid and lipid peroxides. We found clear differences between the oxidative properties of rHbA and rHbF indicating that rHbF is more oxidatively damaging than rHbA at least with regard to lipid damage.

#### Liposome reactions

These experiments used liposomes formed from phosphatidylcholine, containing small amounts of lipid hydroperoxides, which react with hemoproteins as described previously [47]. The lipid oxidation reaction is initially slow, producing a lag phase before ferryl Hb and radical concentrations reach a critical level and a cascade of lipid oxidation starts. Therefore, the longer the lag phase, the less damaging the Hb. Under all conditions tested rHbF was found to be more damaging than rHbA. When reductant (ascorbate) was added to the assay the lag times increased for both rHbA and rHbF but less so for rHbF for both met and oxy Hb. This is consistent with our other data showing the oxidiz ed forms of rHbF being less readily reducible than the adult protein.

#### HPODE reactions

The reaction of Hb with the lipid peroxide HPODE is a complex one that leads to degradation of the heme and destruction of conjugated dienes of the lipid [64]. Our experiments monitoring heme bleaching followed a similar pattern to our data on lipid oxidation. Again, rHbF was more aggressive suggesting it has the potential to be more damaging in an environment containing lipids.

### Oxidative damage to primary cell lines

Cytotoxicity of extracellular Hb molecule through its redox cycling reactions and generation of reactive oxidative species is well documented [65,66]. HUVECs were treated with rHbA and rHbF (oxyHb and metHb). General levels of cellular damage were measured by LDH release and oxidative stress was monitored by the induction of the defence protein SOD.

The addition of 10 mU GOX to cellular medium generates H_2_O_2_, a potent oxidizing agent that creates substantial oxidative stress [67]. This positive control increased both LDH release and SOD induction. The met forms of the recombinant proteins induced oxidative stress, but did not increase overall cellular damage. In contrast, the oxy forms increased both oxidative stress and cellular damage, suggesting that the superoxide produced during the autoxidation process was a significant contributory factor in the heme-induced damage.

The combined Hb and GOX treatment data showed increased cellular damage and oxidative stress compared with rHb only, but decreased or similar damage compared with GOX alone, i.e. the effects of GOX and rHb are not additive in this system. This reflects the ‘Janus face’ of Hb interactions with added peroxide in cellular systems. Hb acts as a peroxidase via the formation of intermediate ferryl iron and protein bound free radicals [47]. In certain cases Hb can exacerbate GOX induced cellular damage due to the reactivity of these oxidizing species [7], but in others damage can actually decrease if these Hb ferryl and radical species are less reactive than other cellular targets of peroxide [68]. This protective effect is seen most clearly when Hb is added to peroxide-treated cells in the presence of haptoglobin as haptoglobin binds tightly to Hb decreasing its oxidative reactivity, without diminishing its peroxidase activity [63], in effect acting as a ‘safe’ peroxide removal mechanism. Reductants can also modulate Hb oxidative cytotoxicity. Physiological levels of ascorbate in the plasma, typically in the range of 50 µM, added to cellular medium reduced the cytotoxic damage from both rHb (adult and fetal) with or without the addition of GOX, in line with previous investigations [69,70].

A direct-paired comparison (Supplementary Table S1) revealed very few significant differences between adult and fetal rHb. Where a difference was found, rHbF was found to be less cytotoxic than rHbA. The reactivity of fetal and adult Hb seem to vary with the assay system. In cells (the present paper) and when added to DNA [25], fetal Hb seems somewhat less damaging. However, when added to lipids (the present paper), reverse is the case and fetal Hb is a more aggressive oxidant.

## Conclusion

Recombinant human adult and fetal Hb are both suitable starting materials for producing a HBOC. While the core reactivity or rHbA and rHbF differ, the direction of difference is sometimes in favor of fetal and sometimes in favor of adult. In most cases, additional mutations and/or the subsequent treatment of the protein to enhance vascular lifetime (PEGylation, cross-linking, encapsulation) will likely have a larger effect than the original difference between the β and γ chains.

## Supporting information

**Figure S1.** SDS PAGE electrophoresis for recombinant HbA and HbF. rHbA or rHbF (both 9uM) were subjected to electrophoresis on a 12% Mini Protean TGX Stain Free Gel (BioRad) at 170 V for 30 min. The gel was exposed to UV light for 2 min before visualising. Lanes 2 and 5 represent rHbA and rHbF after purification using CM-Sepharose column, lanes 3 and 6 represent the proteins in the same order after purification using Q-Sepharose column and lanes 4 and 7 after the HisTrap HP column). M = Hb monomer, D = Hb dimer.

**Figure S2.** HPLC analysis for purified recombinant HbA and HbF. Reverse phase HPLC analysis of rHbA (green-280 nm, red-400 nm) or rHbF (blue-280 nm, orange-400 nm) after purification. Heme (iron-protoporphyrin IX) elutes at 13.5 min (off scale for 400 nm). Hb-β elutes at 22.35 min, with both Hb-α and Hb-γ co-eluting at 24.15 min. Other heme-like compounds absorbing at 400 nm are less than 0.3 % of the heme concentration based on the integration of peaks at 400 nm. rHbA and rHbF signals are offset by 10 and 30 milli-AU for clarity.

**Figure S3.** Oxygen binding fractional saturation curves for purified recombinant HbA and HbF. DeoxyHb was titrated with oxygen for rHbA (red circles) and rHbF (blue circles). Data was fitted to a sigmoidal curve based on the Monod-Wyman-Changeux model of the allosteric binding of oxygen to Hb (solid lines). Dotted lines represents the data for the oxygen binding to Hb following PEGylation (red for rHbA and blue for rHbF).

**Table S1.** Student t-tests were performed to compare the cytotoxicity of HbA and HbF on HUVEC cells.

## Abbreviations

DAMP: damage-associated molecule pattern molecule
GOX: glucose/glucose oxidase system
Hb: hemoglobin
HBOC: Hb-based oxygen carrier
HPODE: 13S-hydroperoxy-9Z,11E-octadecadienoic acid
HUVEC: human umbilical vein endothelial cell
LDH: lactate dehydrogenase
rHbA: recombinant adult Hb
rHbF: recombinant fetal Hb
SOD: superoxide dismutase
XIC: extracted ion chromatogram
