# Supplementary material for: Comparison of the oxidative reactivity of recombinant fetal and adult human hemoglobin: implications for the design of hemoglobin-based oxygen carriers
Source: Biosci Rep. 2018 Jul 3;38(4):BSR20180370. doi: 10.1042/BSR20180370 (PMC6028758; doi:10.1042/BSR20180370)
Supplement: Supplementary file 1 [file bsr20180370_Supp1.pdf]

COMPARISON OF THE OXIDATIVE REACTIVITY OF RECOMBINANT FETAL AND ADULT HUMAN HEMOGLOBIN: IMPLICATIONS FOR THE DESIGN OF HEMOGLOBIN-BASED OXYGEN CARRIERS

Supplementary Data

Assay	Experimental treatment HbA vs HbF	p-value
SOD	metHb	0.06652
	metHb + asc	0.7489
	metHb + GOX	0.01406
	metHb + GOX + asc	0.5306
	oxyHb	0.1066
	oxyHb + asc	0.5786
	oxyHb + GOX	0.9345
	oxyHb + GOX + asc	0.8074
LDH	metHb	0.2944
	metHb + asc	0.2391
	metHb + GOX	0.03856
	metHb + GOX + asc	0.08145
	oxyHb	0.9352
	oxyHb + asc	0.5125
	oxyHb + GOX	0.1349
	oxyHb + GOX + asc	0.2998

Table S1. Student t-tests were performed to compare the cytotoxicity of HbA and HbF on HUVEC cells.

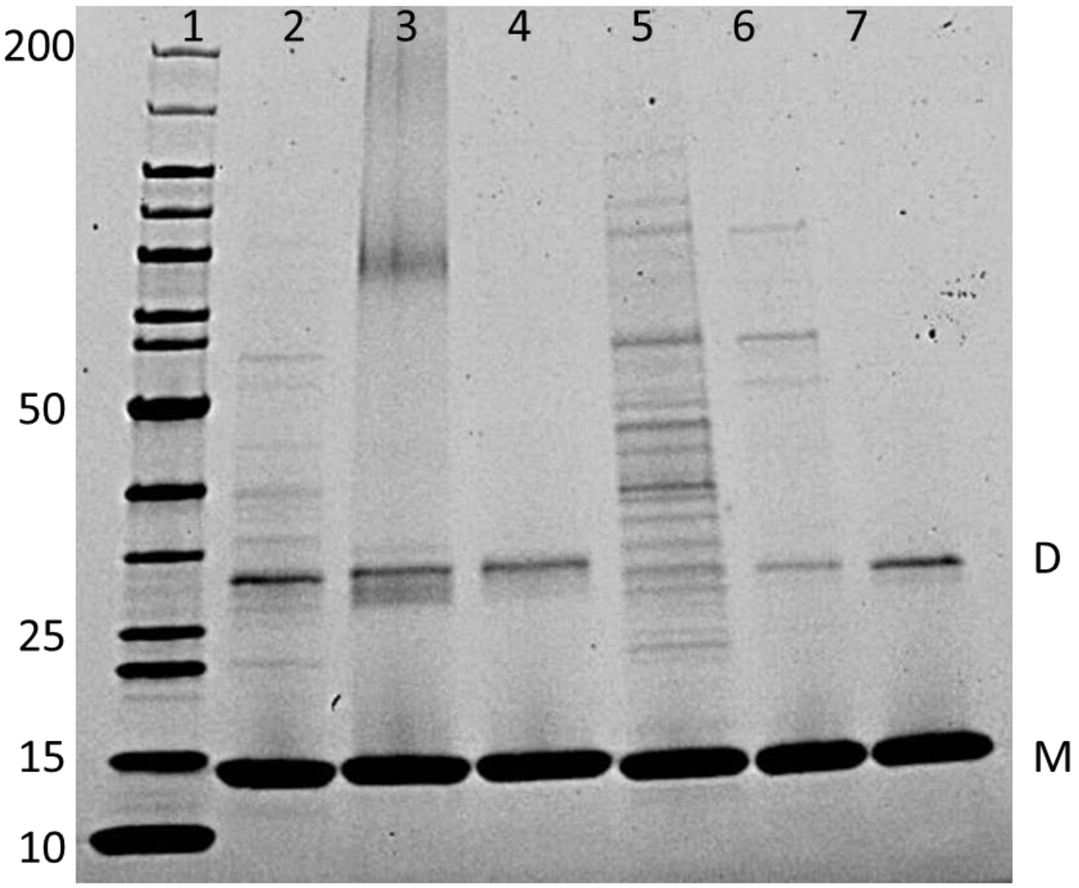

Figure S1. SDS PAGE electrophoresis for recombinant HbA and HbF.

rHbA or rHbF (both 9uM) were subjected to electrophoresis on a 12% Mini Protean TGX Stain Free Gel (BioRad) at 170 V for 30 min. The gel was exposed to UV light for 2 min before visualising. Lanes 2 and 5 represent rHbA and rHbF after purification using CM-Sepharose column, lanes 3 and 6 represent the proteins in the same order after purification using Q-Sepharose column and lanes 4 and 7 after the HisTrap HP column). M = Hb monomer, D = Hb dimer.

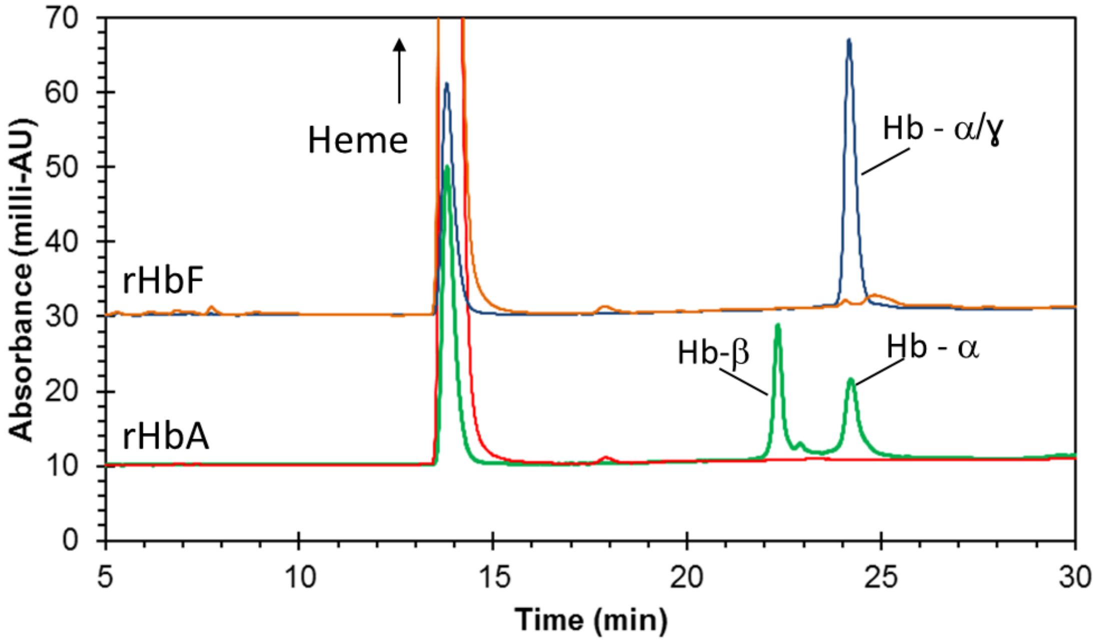

Figure S2. HPLC analysis for purified recombinant HbA and HbF.

Reverse phase HPLC analysis of rHbA (green-280 nm, red-400 nm) or rHbF (blue-280 nm, orange-400 nm) after purification. Heme (iron-protoporphyrin IX) elutes at 13.5 min (off scale for 400 nm). Hb-β elutes at 22.35 min, with both Hb-α and Hb-γ co-eluting at 24.15 min. Other heme-like compounds absorbing at 400 nm are less than 0.3 % of the heme concentration based on the integration of peaks at 400 nm. rHbA and rHbF signals are offset by 10 and 30 milli-AU for clarity.

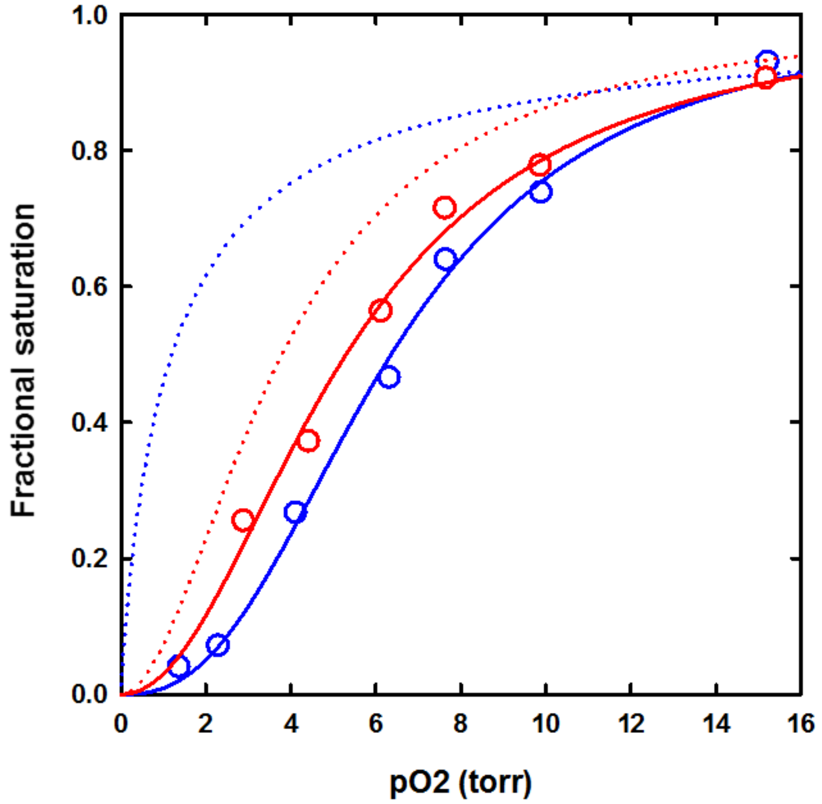

Figure S3. Oxygen binding fractional saturation curves for purified recombinant HbA and HbF.

DeoxyHb was titrated with oxygen for rHbA (red circles) and rHbF (blue circles). Data was fitted to a sigmoidal curve based on the Monod-Wyman-Changeux model of the allosteric binding of oxygen to Hb (solid lines). Dotted lines represents the data for the oxygen binding to Hb following PEGylation (red for rHbA and blue for rHbF).
